# Spatial patterns of vegetation carbon sinks and sources dataset in Central Asia

**DOI:** 10.1016/j.dib.2020.106200

**Published:** 2020-08-20

**Authors:** Zhi Li, Yaning Chen, Qifei Zhang, Yang Li

**Affiliations:** a State Key Laboratory of Desert and Oasis Ecology, Xinjiang Institute of Ecology and Geography, Chinese Academy of Sciences, Urumqi 830011, China; b University of Chinese Academy of Sciences, Beijing 100049, China; c Nuclear and Radiation Safety Center, Beijing 100082, China

**Keywords:** Net ecosystem productivity (NEP), Net primary productivity (NPP), Land use/cover change, Central Asia

## Abstract

This dataset includes land use/cover change data with a spatial resolution of 300 m, net ecosystem productivity data based on the monthly grid data of the temperature and precipitation series data from the Climatic Research Unit, terrestrial net primary production data from MOD17 of the Central Asia that underlies the article entitled “Spatial patterns of vegetation carbon sinks and sources under water constraint in Central Asia”. We explain the details of the dataset, the data harmonization procedures, and the spatial coverage. We also provide the validation result of NPP data from MOD17. We unified the spatiotemporal resolution of these data from different sources, based on re-sampling (nearest neighbor interpolation) and re-classification techniques, and combined the data from the different source datasets to form comprehensive records.

**Specifications Table**

**Table:** 

**Subject**	**Environmental Science**
**Specific subject area**	Nature and Landscape Conservation
**Type of data**	Figure Table
**How data were acquired**	All data were compiled via open address of global dataset. Instruments: software Model and make of the instruments used: Matlab 2013a URL: http://cn.mathworks.com/products/matlab/
**Data format**	Raw Analyzed
**Parameters for data collection**	We describe the methods of harmonization and validation used to build this harmonized dataset.
**Description of data collection**	The monthly grid data of the temperature and precipitation series from 2000-2015, with a spatial resolution of 0.5 degrees, were collected from the Climatic Research Unit. The land use/cover change data with a spatial resolution of 300 m were collected from http://maps.elie.ucl.ac.be/CCI/viewer/
**Data source location**	City/Town/Region: Central Asia Country: Kazakhstan, Kyrgyzstan, Tajikistan, Turkmenistan, and Uzbekistan
**Data accessibility**	With the article
**Related research article**	Author's name: Zhi Li, Yaning Chen, Qifei Zhang, Yang Li Title: Spatial patterns of vegetation carbon sinks and sources under water constraint in Central Asia Journal: Journal of Hydrology DOI: 10.1016/j.jhydrol.2020.125355

**Value of the Data**

Researchers who are focus on the ecological issues in Central Asia can benefit from these data. This dataset can be used as a baseline for a separate country or other small area.
These data could be used for further fully simulate energy, water, and carbon exchanges via earth system models in Central Asia.

## 1 Data

The monthly grid data of the temperature and precipitation series from 2000–2015 (Supplementary Raw data_temperature and Supplementary Raw data_precipitation), with a spatial resolution of 0.5 degrees in Central Asia were collected from the Climatic Research Unit (http://www.cru.uea.ac.uk/data/). The land use/cover change data with a spatial resolution of 300 m were collected from http://maps.elie.ucl.ac.be/CCI/viewer/. The global 1 km NPP datasets (2000–2015) were from MOD17. Net ecosystem production (NEP) data were obtained from the difference value between net primary production and heterotrophic (soil) respiration (R_H_).

## 2 Experimental Design, Materials, and Methods

### 2.1 Net ecosystem production (NEP) algorithm

Net ecosystem production (NEP) can describe the carbon source/sink of ecosystem qualitatively and quantitatively. It is defined as the difference between net primary production and heterotrophic (soil) respiration, and represents the total amount of organic carbon in an ecosystem available for storage [1,2]. It is an important scientific index for the estimation of global and regional carbon balance. The formula is as follows:

(1) $$\text{NEP}=\text{NPP}-R_{H}$$

(2) $$R_{H}=0.22\;\times \left[\text{Exp}\left(0.0913T\right)+\text{Ln}\left(0.3145P+1\right)\times 30\times 46.5\%\right]$$

where NPP is the net primary production, RH is the soil microbial respiration, T is the monthly mean temperature (C), P is the monthly precipitation (mm).

**Fig. 1:**
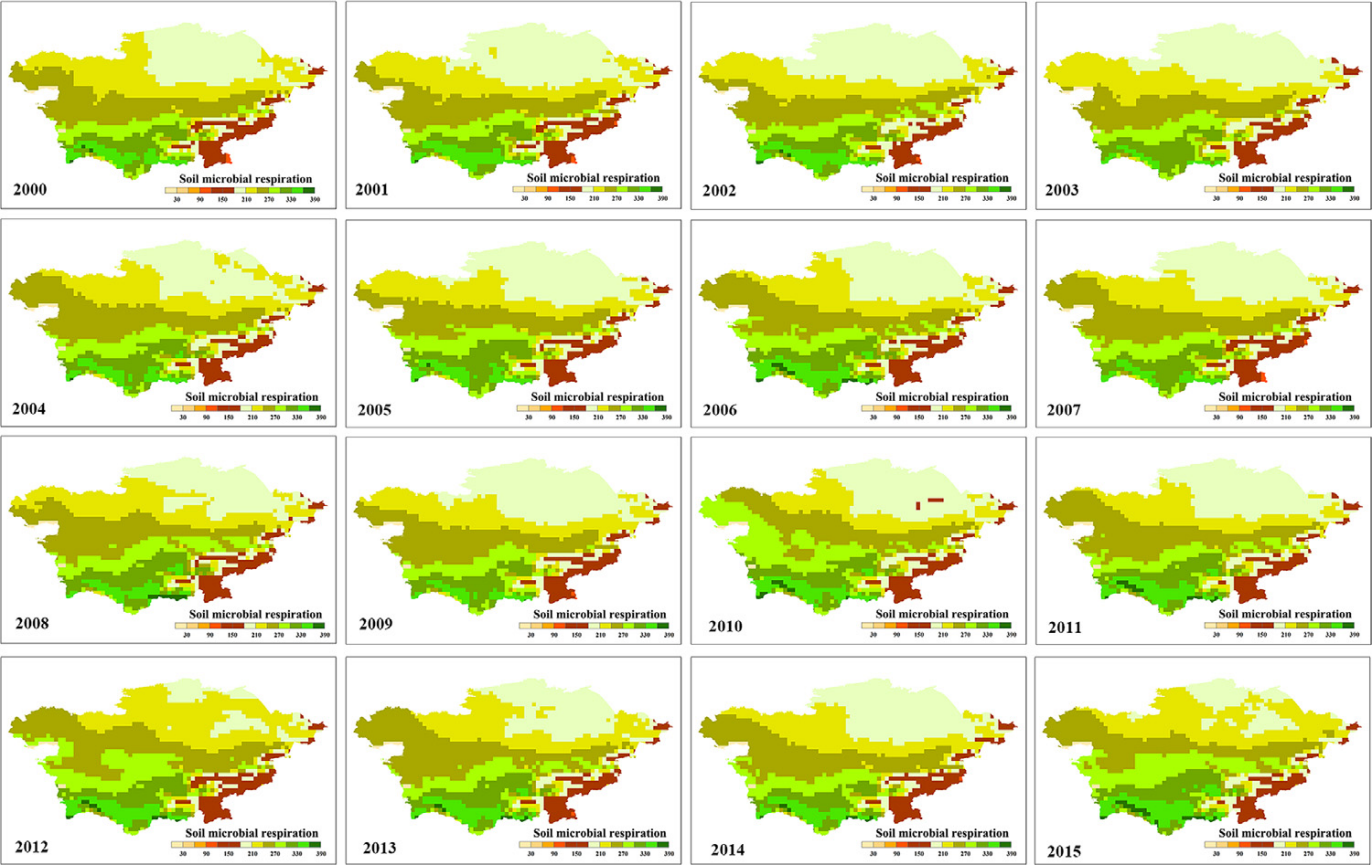
Spatial distribution of annual RH (soil microbial respiration)

**Fig. 2:**
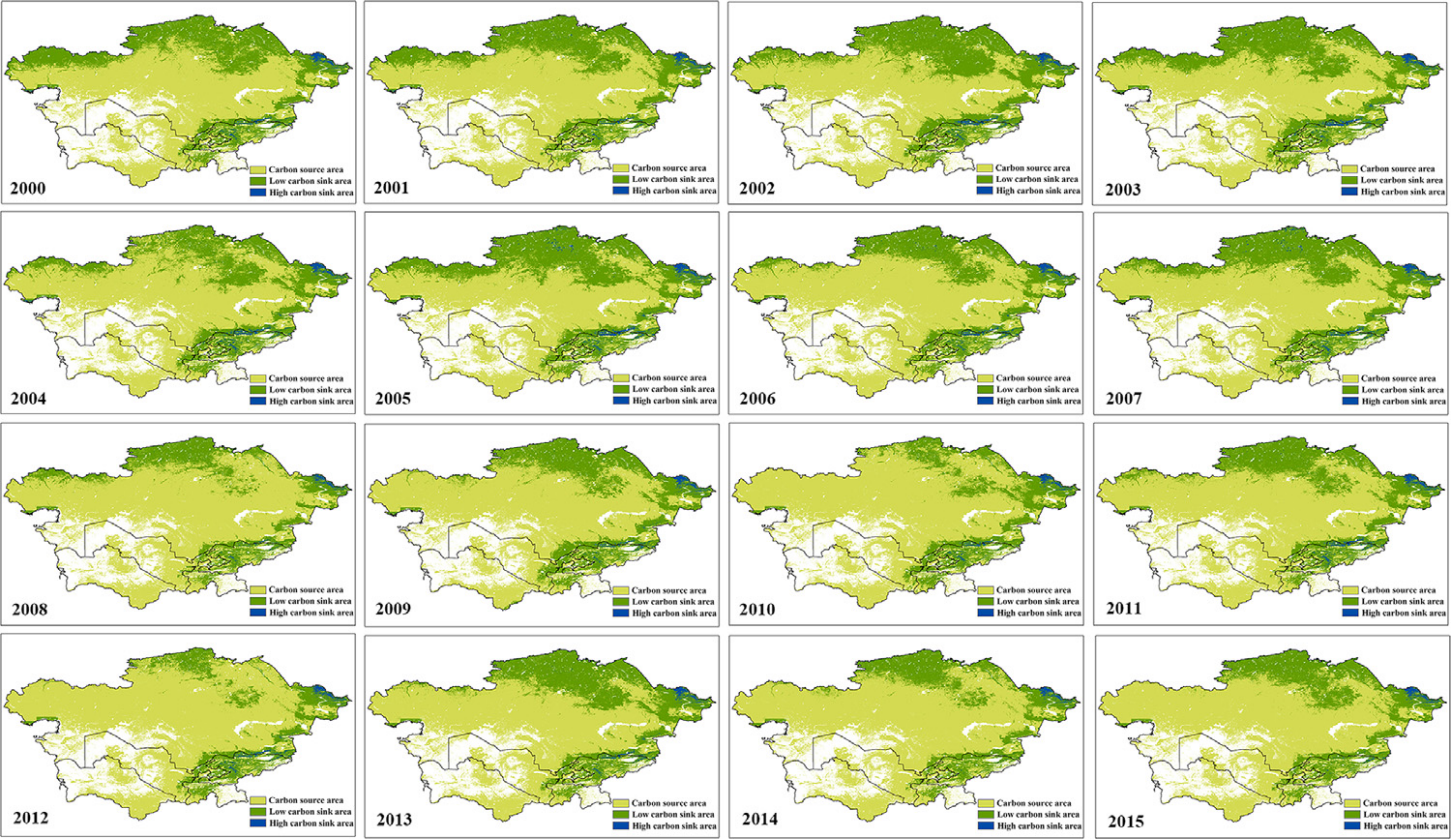
Spatial distribution of annual NEP

Fig. 1 is the spatial distribution of annual RH over 2000–2015, which calculated from the formula 2 (Supplementary modelled data_Rn_resample). Fig. 2 is the spatial distribution of annual NEP over 2000–2015, which calculated from the formula 1 (Supplementary file of NEP_calculation.m).

### 2.2 Validation of NPP data

The arid region of northwest China which is near the Central Asia, is located in the hinterland (35°–50° N, 73°–106° E) of the Eurasia continent. It has similar climatic and environmental conditions. Excluding several sites that have prolonged missing data, the data used in this study are collected from 38 ground-based meteorological sites in the region operated by the China Meteorological Administration, which have complete records of almost all the climatic factors from 2000-2014. We used the total of 570 points in the arid regions of northwest China to validate the NPP algorithm and NPP data from MOD17 based on various regressions (multiple regression, principal component analysis, ridge regression and partial square regression). Total speaking, terrestrial NPP estimates allows the use of MODIS in arid and semi-arid areas and reflects vegetation growth and distribution [3]. The multiple regression model is considered to be a suitable model for the simulation of NPP in arid regions, and the simulation results have a strong correlation with MOD17A3 products. The table 1 is the distribution of the 38 ground-based meteorological sites.

**Table 1:** Sites distribution

Site No.	Sites	Longitude	Latitude	Site No.	Sites	Longitude	Latitude
51053	Habahe	86.40	48.05	51701	Torugart	75.40	40.52
51059	Jeminay	85.87	47.43	51711	Akqi	78.45	40.93
51068	Fuhai	87.47	47.12	51716	Bachu	78.57	39.80
51076	Altay	88.08	47.73	51720	Keping	79.05	40.50
51087	Fuyun	89.52	46.98	51765	Tikanlik	87.70	40.63
51133	Tacheng	83.00	46.73	51804	Tashkurghan	75.23	37.77
51186	Qinghe	90.38	46.67	51811	Shache	77.27	38.43
51241	Tuoli	83.60	45.93	51839	Minfeng	82.72	37.07
51288	Beitashan	90.53	45.37	51855	Qiemo	85.55	38.15
51330	Wenquan	81.02	44.97	51931	Yutian	81.65	36.85
51365	Caijiahu	87.53	44.20	52101	Balitang	93.05	43.60
51379	Qitai	89.57	44.02	52203	Hami	93.52	42.82
51437	Zhaosu	81.13	43.15	52446	Dingxin	99.52	40.30
51467	Baluntai	86.30	42.73	52546	Gaotai	99.83	39.37
51477	Dabancheng	88.32	43.35	52633	Tuole	98.42	38.80
51542	Bayanbulak	84.15	43.03	52661	Shandan	101.08	38.80
51567	Yanqi	86.57	42.08	52674	Yongchang	101.97	38.23
51633	Baicheng	81.90	41.78	52679	Wuwei	102.67	37.92
51642	Luntai	84.25	41.78	53602	Alxa Zuoqi	105.67	38.83

**Table 3:** Land use/cover areas (km^2^)

	2000	2005	2010	2015
Cultivated land	544288.90	548946.25	549387.92	645880.02
Grassland	1089379.95	1094184.60	1093251.22	1093804.40
Forestland	310852.44	326098.36	343690.39	249810.94
Shrubland	283912.38	284191.79	284466.13	284732.46
Sparse vegetation	708980.21	699830.32	710163.03	715582.12
Urban areas	3130.92	6057.44	7366.32	8971.28
Water/Ice	144381.30	134687.87	126332.93	122033.06
Bare areas	943192.89	934121.34	913459.99	907304.69

### 2.3 Land use/cover change data

Based on the land use/cover classification data from 2000 to 2015 (Supplementary modelled data_LUCC), we resampled the original 36 categories into 8 categories. Then, we got the distribution and variations of carbon sources/sinks in different vegetation types. The areas of land use/cover change in the year 2000, 2005, 2010 and 2015 are as follows:

## Declaration of Competing Interest

None.

## Appendix Supplementary materials

Image, application 1

Image, application 2
